# Late-Onset Idiopathic Generalized Epilepsy Manifesting With De Novo Late-Onset Absence Status Epilepticus After COVID-19 Infection: A Case Report

**DOI:** 10.7759/cureus.74618

**Published:** 2024-11-27

**Authors:** Mao Otake, Go Taniguchi, Hideo Kato, Yuichiro Fuji, Chihiro Nakata, Eiji Nakagawa

**Affiliations:** 1 Department of Epileptology, National Center Hospital, National Center of Neurology and Psychiatry, Tokyo, JPN

**Keywords:** absence status epilepticus, covid-19, electroencephalogram, generalized tonic-clonic seizure, idiopathic generalized epilepsy

## Abstract

Herein, we present a case of idiopathic generalized epilepsy (IGE) manifesting as de novo late-onset absence status epilepticus (ASE) following mild coronavirus disease 2019 (COVID-19). A woman in her 40s presented with persistent 3-5.5 Hz generalized spike-wave complexes (SWCs) on electroencephalography (EEG). She experienced a generalized tonic-clonic seizure (GTCS) 10 days after the COVID-19 infection. She was diagnosed with epilepsy at 40 years of age, after which she was started on levetiracetam (LEV) 1,000 mg/day. After the medication was started, she experienced three other GTCSs. We performed a long-term video EEG of the patient, leading to a diagnosis of late-onset IGE with de novo late-onset ASE. She achieved seizure freedom for over a year with a combination of valproic acid (VPA) and lamotrigine (LTG), although her EEG continued to demonstrate persistent generalized SWCs. This case suggests that late-onset IGE accompanied by de novo late-onset status epilepticus may have been triggered by COVID-19. Persistent neuroinflammation may be reflected in persistent EEG abnormalities, even after epileptic seizures are well controlled with medications. LTG and VPA are believed to be effective for clinical management, and EEG is an essential modality for recording epileptic activity in outpatient settings.

## Introduction

The association between coronavirus disease 2019 (COVID-19) and the onset of epilepsy has previously been documented; however, the mechanisms underlying this association are yet to be fully elucidated [1]. One report suggested that non-hospitalized patients were also at an increased risk of seizures and epilepsy after COVID-19 [2]. Although many cases of seizures and temporal lobe epilepsy following COVID-19 have been reported [3,4], cases of post-COVID generalized epilepsy have not yet been reported.

One prior study [5] suggested that de novo late-onset absence status epilepticus (ASE) could be diagnosed using the following criteria: (1) certain seizure types (generalized tonic-clonic seizure (GTCS) and ASE); (2) a specific EEG pattern (generalized 3 Hz spike-wave complex (SWC) with interictal generalized epileptiform discharges and normal background activity); (3) normal neurological examination, cognition, and neuroimaging findings; and (4) a lack of a history of epileptic seizures. ASE is classified as generalized nonconvulsive status epilepticus (NCSE) without coma according to the current International League Against Epilepsy classification [6]. Our case is consistent with these criteria.

Herein, we present a case of idiopathic generalized epilepsy (IGE) manifesting with de novo late-onset ASE following mild COVID-19.

## Case presentation

A right-handed woman in her 40s presented with four episodes of GTCS during her lifetime. Her medical history was unremarkable, and she had no history of febrile or afebrile seizures. Further, she had no family history of epilepsy or febrile seizures. The patient was diagnosed with mild COVID-19 and did not require hospitalization or supplemental oxygen therapy. Ten days after the initiation of COVID-19 infection, the patient experienced her first episode of GTCS, losing consciousness approximately one hour before the first seizure episode. The patient had unclear responses to what her husband said and purposely strolled around the house. Afterward, she suddenly screamed and collapsed forward. Her arms and legs became rigid and began to jerk symmetrically for approximately three minutes. The patient was transported to Hospital A, where she was alert and conscious. During a 30-minute EEG, SWCs were repetitively and episodically observed without any clinical features. Brain magnetic resonance imaging (MRI) and laboratory tests were subsequently performed, yielding no abnormalities. The patient was diagnosed with epilepsy based on EEG findings and was administered levetiracetam (LEV; 1,000 mg). She was discharged on the day of admission, and outpatient follow-up was started. Her seizures were effectively managed with medication; however, generalized SWC persisted on EEG. The patient had no psychiatric symptoms or impaired consciousness. Consequently, the dose of LEV was increased to 2,000 mg, and 200 mg of lamotrigine (LTG) was initiated. Despite medical adjustments, SWCs persisted.

Ten months after the first seizure, the patient experienced a second seizure. A few hours before the seizure, the patient exhibited unusual behavior, including impaired consciousness. The patient suddenly wandered away while shopping and spoke incomprehensibly to a stranger; however, she had no memory of these episodes. Afterward, she screamed while walking and fell face-first to the ground. Subsequently, her eyes deviated upward, and her arms and legs became rigid and symmetrically jerked, which was identified as the second episode of GTCS. The patient gradually regained consciousness after the seizure when her husband called an ambulance. The patient visited the emergency department and was discharged on the same day. However, on the same day, she experienced two other GTCSs and visited an emergency department after each episode. The patient was discharged from a third hospital the following day. In response to these episodes, LEV was increased to 3,000 mg at a hospital follow-up visit, but episodic generalized SWC did not improve. The patient was thus referred to our hospital for further evaluation.

**Figure 1:**
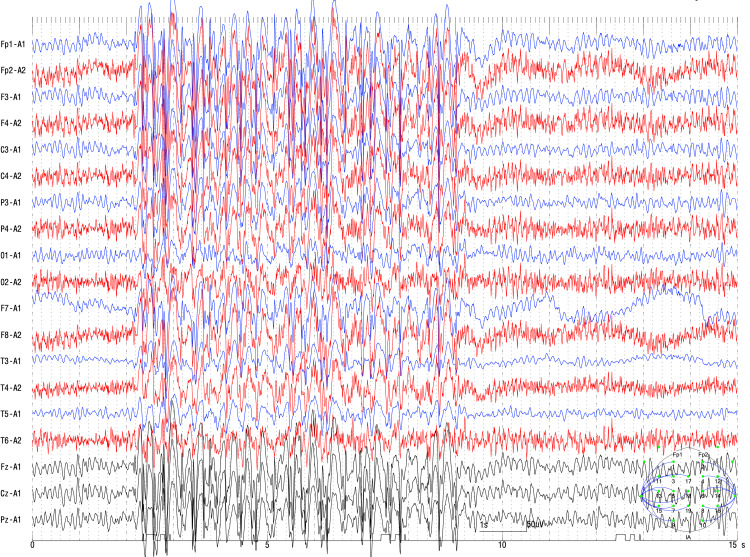
Interictal epileptic discharges (IEDs) characterized by generalized spike-wave complexes (SWCs) The episodic 4-5.5 Hz SWCs persisted as IEDs in both the awake and sleep phases.

We performed long-term video EEG over five days following the discontinuation of 3,000 mg of LEV. Video EEG revealed interictal epileptic discharges (IEDs) characterized by generalized SWCs at a frequency of 4-5.5 Hz (Figure 1).

**Figure 2:**
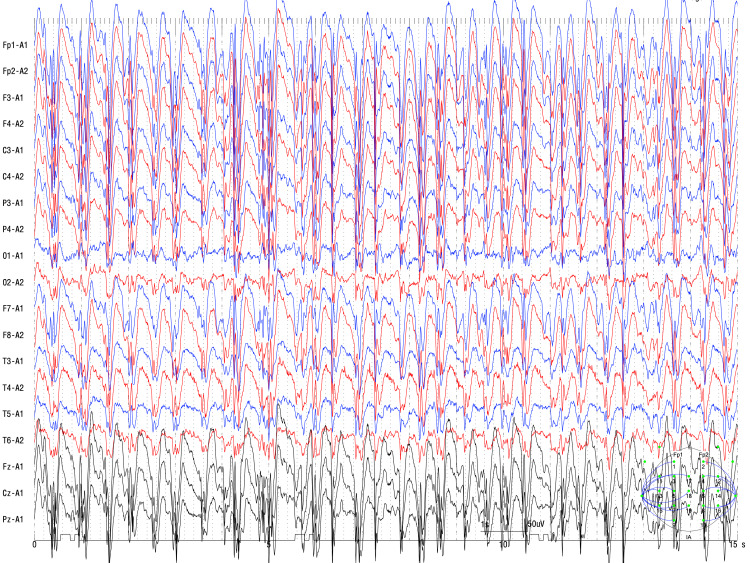
Continuous generalized spike-wave complexes (SWCs) during the impaired conscious phase

Approximately one hour before a seizure was recorded, the patient exhibited a progressive inability to respond to her name or the current date and was unable to perform the serial seven subtraction test. During this period, the EEG exhibited SWCs at a frequency of 2.5-3.5 Hz (Figure 2).

**Figure 3:**
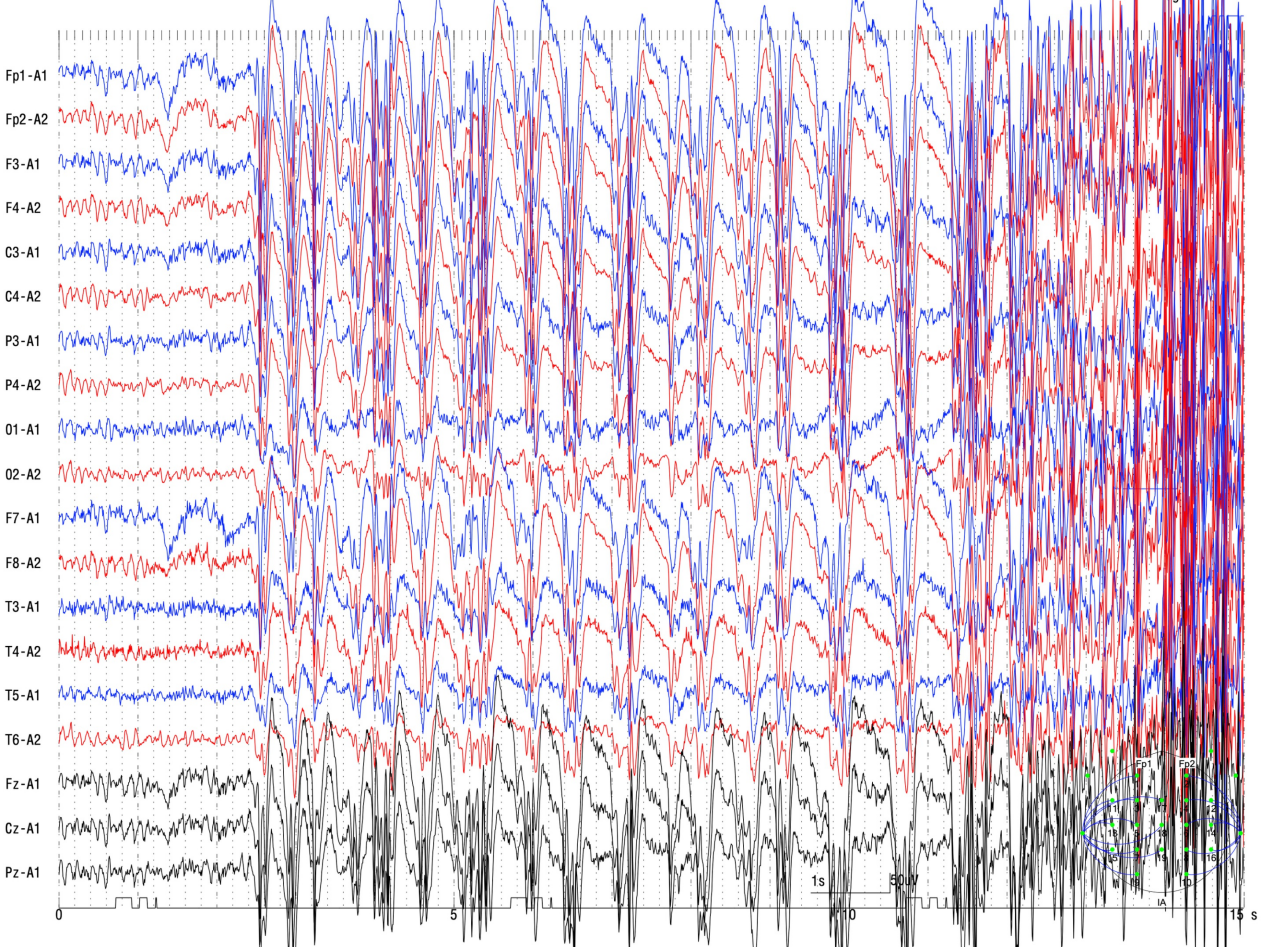
Ictal generalized 3 Hz spike-wave complexes (SWCs) followed by high-voltage polyspikes

She then shouted and experienced a GTCS that lasted for approximately three minutes. No clinical or focal EEG features were observed. Ictal EEG demonstrated continuous 3 Hz SWCs followed by high-voltage polyspikes (Figure 3).

Brain MRI was normal. Cerebrospinal fluid analysis revealed normal antibody titers (Table 1). The Wechsler Adult Intelligence Scale (WAIS)-Ⅳ displayed the following results: Full-Scale Intelligence Quotient (FSIQ), 124; Verbal Comprehension Index (VCI), 128; Perceptual Reasoning Index (PRI), 118; Working Memory Index (WMI), 109; and Processing Speed Index (PSI), 118.

**Table 1:** Cerebrospinal fluid analysis results AMPA, α-amino-3-hydroxy-5-methyl-4-isoxazolepropionic acid; CASPR2, contactin-associated protein-like 2; DPPX, dipeptidyl-peptidase-like protein-6; GABA_B_, anti-gamma aminobutyric acid-B receptor; LGI-1, leucine-rich glioma inactivated 1; NMDAR, anti-N-methyl-D-aspartate receptor

Parameter	Protein (mg/dL)	Glucose (mg/dL)	White cell count	Oligoclonal banding	NMDAR	CASPR2	LGI-1	GABA_B_	AMPA	DPPX
Result	43	53	1/3	(-)	(-)	(-)	(-)	(-)	(-)	(-)
Reference range	10-40	50-75	0	N/A	N/A	N/A	N/A	N/A	N/A	N/A

Based on these results, the patient was diagnosed with IGE. Although the LTG dose was increased to 400 mg in the outpatient setting, IEDs persisted. The risk of seizure recurrence is high, particularly during sleep deprivation. We started administration of 400 mg valproic acid (VPA) after reducing LTG to 200 mg, anticipating an increase in LTG blood levels. The blood concentration of LTG increased from 6.33 to 8.76 μg/mL. At the most recent outpatient follow-up, the patient had not experienced any new seizures; however, generalized SWCs were still observed approximately 10 times during the 30-minute EEG recording. Cognitive ability was preserved despite the persistence of epileptic activity on the EEG.

## Discussion

To our knowledge, this is the first case report of IGE following COVID-19 with the onset of GTCS after infection. All seizures the patient experienced involved tonic movements, followed by clonic movements that terminated simultaneously in both limbs. Sets of 2.5-5.5 Hz generalized SWCs consistently appeared as IEDs. Complications, including encephalitis, were excluded. Based on these findings, the patient was diagnosed with IGE. One report [7] indicated that IGEs could go undiagnosed, particularly when the EEG has asymmetric features. This revealed that some cases of late-onset IGEs may be undiagnosed early in life and could be clinically diagnosed later with epilepsy. In our case, it is still possible that the patient had abnormal EEG findings before experiencing the first GTCS. However, it was impossible to investigate past EEG findings as the patient had never undergone EEG recordings before the first GTCS. However, several detailed, highly reliable interviews with the patient and her family, which precisely pictured her seizure, led us to conclude that she had experienced her first seizure episode after COVID-19 infection.

Regarding clinical presentation, the patient presented with an impaired consciousness phase lasting approximately one hour before the onset of GTCS. During this phase, she was unable to accurately provide her name, identify the date, or perform seven serial subtractions. EEG during the impaired consciousness phase revealed more than 25 SWCs at a frequency of 3-3.5 Hz within a 10-second period, meeting the Salzburg criteria for NCSE. NCSE, which appears later in adults with no history of epilepsy, was first reported by Brigo et al. [5] as a de novo late-onset ASE and could occur along with late-onset IGE. This condition is prevalent in women and can be triggered by benzodiazepine (BZD) withdrawal [8], chronic alcohol use, initiation of drugs, such as antidepressants, metabolic abnormalities, infections, and dehydration [9]. Conversely, Brigo et al. [5] reported nine cases of de novo late-onset ASE without triggering factors. These studies indicate that underlying triggers often initiate de novo late-onset ASE; however, this condition can also occur without any triggers. In our case, the COVID-19 infection was likely the trigger for de novo late-onset ASE, followed by late-onset IGE. 

Tsai et al. [10] previously reported that the percentage of COVID-19 patients with seizures whose CSF tested positive for SARS-CoV-2-PCR was low. Furthermore, a retrospective cohort study of seven patients [11] revealed that non-contrast-enhanced head computed tomography (CT) showed no new abnormalities in any of the investigated cases. Dono et al. [12] further presented that 29.8% and 42.6% of patients who underwent CT and MRI showed abnormalities in cases of SE associated with COVID-19. A previous study [13] also reported that 96 of 197 (48.7%) patients with COVID-19 had epileptiform EEG abnormalities. Another report [14] revealed that more than half of COVID-19 patients had abnormal EEG findings (abnormal background activity in 81% and frontal slow waves in 60%). Our case presented persistent SWCs on EEG, without CSF or brain MRI findings, and we suggest that EEG monitoring is a highly sensitive method for monitoring the state of seizures in COVID-19 patients.

In regards to EEG findings, previous studies using long-term follow-up have presented contradictory results; some have observed rapidly normalized IEDs [5,15], while others have noted their persistence [5,16]. Thomas et al. [15] reported that EEG normalized with intravenous and oral BZD within 72 hours of absence. Conversely, Pro et al. [16] described the case of an 86-year-old patient treated with LTG 200 mg who demonstrated interictal 3-3.5 Hz SWCs on EEG for 14 years.

Regarding treatment, Brigo et al. [5] reported that six patients with de novo late-onset ASE achieved seizure freedom while receiving monotherapy with VPA (83%) or LTG (17%) during a follow-up of three to 24 months. VPA is the first choice for IGE treatment; however, its use in women of reproductive age remains controversial. In one retrospective cohort study targeting women of childbearing age, LEV was considered an initial alternative monotherapy [17]. Moreover, LEV has been suggested to contribute to seizure control in 27% of cases of absence seizures [18]; however, Auvin et al. reported six pediatric patients with aggravation of absence seizures with LEV [19]. LTG has been reported to provide therapeutic outcomes comparable to those of VPA for the typical absence of seizures [20]. Additionally, a previous study indicated that seizure control was achieved in 71.4% of the patients newly diagnosed with typical absence seizures [21]. Regarding combination therapy with VPA and LTG, a treatment response was achieved in 45% of refractory epilepsy cases, surpassing 35% with other combination therapies [22]. Based on a comprehensive literature analysis, we found that combination therapy with LTG and VPA was the most appropriate treatment for our case of late-onset IGE with de novo late-onset ASE.

Infections of the central nervous system (CNS) can trigger acute symptomatic seizures. One prior review [23] showed that traumatic brain injury and CNS infections were associated with a cumulative 10-year risk of unprovoked seizure relapse following the first acute symptomatic seizure of 19% compared to 65% (55-74%) after the first unprovoked seizure. This indicates that the prognosis in our case was worse than that of acute symptomatic seizures, which justifies the long-term prescription of anti-seizure medications.

Finally, we discuss the mechanisms underlying de novo late-onset ASE after COVID-19. Severe acute respiratory syndrome coronavirus 2 (SARS-CoV-2) is believed to infect the CNS via the blood-brain barrier (BBB) [24,25]. Based on previous studies, we propose two potential mechanisms by which SARS-CoV-2 can lead to de novo late-onset ASE: (1) SARS-CoV-2, upon entering the CNS, induces an inflammatory cascade, leading to the production of pro-inflammatory cytokines such as interferon (IFN)-β, IFN-λ1, IFN-γ, interleukin (IL)-2, IL-6, IL-17, CXCL8, CXCL9, and CXCL10, resulting in chronic neuroinflammation [26]; and (2) SARS-CoV-2 binds to the host angiotensin-converting enzyme 2 (ACE2) receptor, suppressing ACE2 expression, increasing angiotensin II levels, and causing electrolyte disturbances [27]. In the present case, no electrolyte abnormalities were observed on admission, suggesting that neuroinflammation may have been more relevant. In the presence of prolonged symptoms following COVID-19 infection, known as long COVID, persistent neuroinflammation has been suggested to be involved in neurological symptoms, such as “brain fog,” headaches, and cognitive impairment [26]. In the present case, the persistence of IEDs may have been associated with persistent neuroinflammation. Furthermore, although cognitive impairment was not clinically observed, the WAIS-IV displayed disparities among the subscales, suggesting that persistent neuroinflammation may cause a deficit in working memory, which was previously reported as a major comorbidity of epilepsy [28].

## Conclusions

In conclusion, our experience with the present case suggests that late-onset IGE accompanied by de novo late-onset ASE may be triggered by infections such as COVID-19. Persistent neuroinflammation may have occurred as a result of persistent IEDs and working memory deficits, even after the epileptic seizures were adequately managed with medication. LTG and VPA are effective clinical management strategies, and long-term prescriptions may be necessary for a better prognosis. EEG is a sensitive and reliable method for diagnosing and monitoring late-onset IGE accompanied by de novo late-onset ASE.
